# The use of the circulating cathodic antigen (CCA) urine cassette assay for the diagnosis and assessment of cure of *Schistosoma mansoni* infections in an endemic area of the Amazon region

**DOI:** 10.1590/0037-8682-0562-2019

**Published:** 2020-09-25

**Authors:** Sergei Rodrigo Magalhães de Sousa, Joyce Favacho Cardoso Nogueira, Isabelle Helena Lima Dias, Álvaro Luan Santana Fonseca, Vivian Favero, Stefan Michael Geiger, Martin Johannes Enk

**Affiliations:** 1 Universidade do Estado do Pará, Programa de Pós-Graduação Strictu Sensu em Biologia Parasitária na Amazônia, Belém, PA, Brasil.; 2 Instituto Evandro Chagas/SVS/MS, Laboratório de Parasitoses Intestinais, Esquistossomose e Malacologia, Secção de Parasitologia, Ananindeua, PA, Brasil.; 3 Pontifícia Universidade Católica do Rio Grande do Sul, Programa de Pós-Graduação em Medicina e Ciências da Saúde, Laboratório de Parasitologia Biomédica, Porto Alegre, RS, Brasil.; 4 Universidade Federal de Minas Gerais, Departamento de Parasitologia, Belo Horizonte, MG, Brasil.

**Keywords:** Schistosoma mansoni, Kato-Katz, Helmintex^®^, POC-CCA, Treatment

## Abstract

**INTRODUCTION:**

Schistosomiasis is a poverty-related disease that affects people in 78 countries worldwide. This study aimed to evaluate the point-of-care circulating cathodic antigen (POC-CCA) test performance using sensitive parasitological methods as a reference standard (RS) in individuals before and after treatment.

**METHODS:**

The RS was established by combining the results of 16 Kato-Katz slides and the Helmintex^®^ method. Positivity rates of the POC-CCA test and Kato-Katz and Helmintex^®^ methods were calculated before treatment and 30 days afterward. Furthermore, the sensitivity, specificity, accuracy, and *kappa* coefficient before treatment were determined by comparing the methods. The cure rate was defined 30 days after treatment.

**RESULTS:**

Among the 217 participants, the RS detected a total of 63 (29.0%) positive individuals. The POC-CCA test identified 79 (36.4%) infections. The evaluation of POC-CCA test performance in relation to the RS revealed a sensitivity of 61.9%, specificity of 74.0%, accuracy of 70.5%, and *kappa* coefficient of 0.33. Out of the 53 remaining participants after treatment, a total of 45 (81.1%) showed egg negative results, and 8 (18.9%) were egg positive according to the RS. A total of 5 (9.4%) egg-positive and 37 (69.8%) egg-negative individuals were positive by the POC-CCA test.

**CONCLUSIONS:**

Our data show that the POC-CCA test has potential as an auxiliary tool for the diagnosis of *Schistosoma mansoni* infection, yielding better results than 16 Kato-Katz slides from three different stool samples. However, the immunochromatographic test lacks sufficient specificity and sensitivity for verifying the cure rate after treatment.

## INTRODUCTION

In 2012, the World Health Assembly adopted a resolution that predicts the interruption of schistosomiasis transmission^1^. In 2015, a total of 118.5 million school-aged children and 100.2 million adults were indicated for preventive chemotherapy with praziquantel^2^. Schistosomiasis affects 78 countries worldwide, and according to the World Health Organization (WHO), preventive chemotherapy is required in 52 endemic countries with moderate to high disease transmission rates. A total of 90 million individuals were treated in 2016 due to the expansion of control interventions^3^.

*Schistosoma mansoni* is the only species found in the Americas, where it is believed that more than 25 million individuals are at risk of infection^4^ ^,^ ^5^. Brazil has the largest area and is responsible for 95% of cases^6^. From 2010 to 2016, regular schistosomiasis control in Brazil revealed a positivity rate of 4.4%^7^. Studies conducted in 2015 and 2018 revealed an estimated 1.5 million infected people^4^ ^,^ ^5^, indicating an overall schistosomiasis prevalence rate of approximately 1.0% in Brazil^5^.

A precise and efficient diagnosis is an important tool for the treatment and control of schistosomiasis^8^ ^,^ ^9^. The currently recommended method for quantitative diagnosis of *S. mansoni* is Kato-Katz (KK) fecal thick-smear slides, which are supposed to detect eggs in infected individuals feces^10^. However, this method has limitations and may underestimate the infection rate by up to 74.0%^8^ ^,^ ^11^ ^-^ ^14^.

In 2007, a new diagnostic method named Helmintex^®^ (HTX), developed specifically for use in areas where the *S. mansoni* egg burden was reduced, showed high sensitivity due to the use of a large amount of feces (30 grams) and several concentration steps that resulted in the isolation of *S. mansoni* eggs through interaction with paramagnetic particles in a magnetic field^15^. Even with a 100% sensitivity for up to 1.3 eggs per gram (EPG) loads^15^, its application on a large scale presented some difficulties; thus, many aspects of the HTX method were optimized, aiming to make it more efficient^16^. The HTX application confirmed this method as a high sensitivity diagnostic tool in endemic areas^14^ ^,^ ^17^ ^,^ ^18^.

Aiming to solve the dilemma for fast and accurate diagnosis of schistosomiasis, a point-of-care (POC) urine test was developed for the detection of schistosome circulating cathodic antigen (CCA). According to data presented in the manufacturer’s manual of the POC-CCA test, the sensitivity rate may vary from 70% to 100%, depending on the intensity of infection^19^. When the POC-CCA test was compared with the KK method, it was reported that a single urine test showed a sensitivity equivalent to that of six^20^ or nine KK slides^21^. However, recent studies on the performance of POC-CCA also showed controversial results, with reduced accuracy and elevated false negative or false positive rates, particularly in low prevalence areas^22^ ^,^ ^14^ ^,^ ^17^ ^,^ ^18^.

The WHO and the Department of Control of Neglected Tropical Diseases in 2015 suggested the use of the POC-CCA test in endemic countries, along with the KK method, for monitoring and evaluation of control programs, whose goal is the elimination of schistosomiasis as a public health problem^2^. A published review^18^ on the use of KK as an RS to evaluate the performance of the POC-CCA test for the diagnosis of *S. mansoni* infections showed that most of the studies were conducted in Africa. To date, ten studies on POC-CCA performance for the diagnosis of intestinal schistosomiasis have been conducted in Brazil. Only a few of them used the HTX method as an RS^14^ ^,^ ^17^. Hence, there is an urgent need to better evaluate the performance of the POC-CCA test, using more sensitive parasitological methods as RSs, such as the KK technique and modified HTX methods. Therefore, the present study aimed to evaluate the POC-CCA test performance using more sensitive parasitological methods as reference standards (RSs) among individuals before and after treatment.

## METHODS

### Study area and population

The present study was conducted from March to October 2014 in the community of Paxiba, municipality of Turiaçú, Maranhão State in Brazil, located 152 km from the capital São Luis. It is part of the Amazon region characterized by a tropical climate, with temperatures ranging from 16ºC to 36.4ºC and an annual average rainfall between 191.9 mm and 218.2 mm^23^.

All 235 community residents were invited to participate in this study. A sample size calculation was not necessary because the entire community was enrolled. Previous surveys conducted by the Brazilian Schistosomiasis Control Program reported a positivity rate of 5.0%.

To be enrolled in the present study, each participant had to deliver stool and urine samples. In addition, children younger than 2 years were excluded.

### Biological sample collection procedures

Among the 217 participants, one morning urine sample and three stool samples were collected on consecutive days at two time points: before treatment and 30 days afterward. It is important to note that only egg-positive individuals were re-examined 30 days after treatment. All biological samples were identified, stored in properly cooled cases, and transported to the Instituto Evandro Chagas SVS/MS. All laboratory procedures were carried out at the Laboratório de Parasitoses Intestinais Esquistossomose e Malacologia, located at the Instituto Evandro Chagas SVS/MS. 

### Kato-Katz method (Katz et al., 1972)

A commercial KK kit (HelmTest; Biomanguinhos, Brazil) was used to prepare slides with fecal smears, according to the manufacturer’s instructions.

A total of 16 KK fecal thick smears were prepared: twelve from the first fecal sample, two from the second, and two from the third. The 12 slides from the first sample summed up to 500 mg of examined fecal matter. Together with the slides from samples two and three, a total of approximately 667 mg of feces was analyzed.

EPG values were calculated based on the number of eggs counted on 16 slides from different samples.

The HTX test was also performed using the first sample. The remaining biological samples were frozen and stored at the Biobank of the Parasitology Section of Instituto Evandro Chagas -Pará State.

### Helmintex^®^

Described by Teixeira *et al*. (2007)^15^ and modified by Favero *et al.* (2017)^16^, the HTX method was specifically developed for *S. mansoni* egg detection. This method consists of concentration steps that aim to select eggs among sediments by applying paramagnetic beads that bind to the schistosome eggshell. After this process, a magnetic field is applied, and the eggs can be separated from the remaining sediment. The final material was added to a 3% ninhydrin solution and spread on filter paper to quantify the eggs by reading under a microscope. In the present study, an average of five filters was examined per sample. Eggs were identified correctly following the proposed criteria based on egg elements such as shape, presence of spike, approximated size, well-defined shell, space between the miracidium and shell, and purple color of the miracidium^21^. 

### POC-CCA

The urine-CCA cassette is recommended for the qualitative detection of an active *Schistosoma* infection, as it is more specific for *S. mansoni* infections. In the present study, the first version of the test (lot number: 50182) was used, provided by the Brazilian Ministry of Health from Rapid Medical Diagnostics, Pretoria, South Africa.

Only one drop of first morning urine was required for the examination. According to the manufacturer’s recommendation, one drop of buffer was added^19^. The test result was reported 20 minutes after adding the buffer to the samples. All the results of the immunochromatographic test were interpreted as positive, considering the development of a second pink line parallel to the control line; otherwise, the test result was considered negative, according to the manufacturer’s recommendations^19^. It is important to note that a weak pink line, classified as a ‘trace’ result, was included in the analysis, first as a positive result and second as a negative result. Three experienced and properly trained laboratory staff analyzed the POC-CCA test to ensure quality test results.

### Reference standard

The RS was composed of a total of 16 KK slides and HTX method analysis to maximize the detection of egg-positive individuals infected with *S. mansoni*. All positive individuals confirmed by the RS were classified as true positives.

### Statistical analysis

Statistical tests were performed using the program OpenEpi version 3.01 by Epidemiologic Statistics for Public Health (https://www.openepi.com/Menu/OE_Menu.htm updated in 2013). The results were paired in 2 × 2 tables with 95% confidence intervals (CIs). The rates of positivity, sensitivity, specificity, positive and negative predictive values, and accuracy were calculated to verify the performance of POC-CCA compared to the RS. The kappa index was calculated to evaluate the concordance between the tests in analysis and RS, following the classification criteria recommended by Landis and Koch (1977)^24^, with concordance values considered as bad (<0.20), weak (0.21-0.40), moderate (0.41-0.60), good (0.61-0.80), and excellent (>0.81).

### Ethical considerations

The study was part of a multicenter project, with participants from the States of Pará, Minas Gerais, and Rio Grande do Sul. The project was submitted to the ethics committee and approved (CAAE: 21824513.9.3001.0019). All participants were informed about the objectives and invited to participate voluntarily, and enrolled individuals signed the consent form. All individuals with positive test results detected according to the RS were treated with praziquantel, following the guidelines of the Brazilian Ministry of Health, with 60 mg/kg for children and 50 mg/kg for adults^9^.

## RESULTS

### Positivity rate by RS

After applying the exclusion criteria, of 235 individuals invited to participate in this study, only 217 remained. Among those, 111 (51.1%) were males and 106 (48.9%) were females. In relation to age, 59 participants were aged 21-40 years, while 20 participants were older than 60 years (Supplementary Table 1). 

The RS detected a total of 63 individuals with positive results, yielding a positivity rate of 29.0% among the 217 participants. Based on the number of positives and positivity rate in relation to sex, 38 (34.2%) males were infected and 25 (23.6%) females were egg-positive.

The positivity rate by age group determined by the RS was highest among individuals aged 11-20 years (n=26). 

### Positivity rate by Kato-Katz and Helmintex^®^ methods

The analysis via the KK technique using 16 slides of different samples among the 217 participants showed that 31 (14.3%) individuals were *S. mansoni* egg positive. Using a single KK slide, a total of 12 (5.5%) egg-positive individuals were detected, which increased further to 17 (7.8%) after reading two slides. The parasitic load assessed via 16 slides from different samples revealed that all the participants had a low parasite load, with less than 100 EPG of feces.

In relation to the HTX method, a total of 53 (24.4%) egg-positive individuals were identified.

### Positivity rate by POC-CCA

Apart from the parasitological examination, a rapid urine test was applied for the detection of CCA. The POC-CCA test identified 79 infected individuals, resulting in a positivity rate of 36.4%. Based on the number of positives and positivity rate in relation to sex, a total of 48 (60.8%) males and 31 (39.2%) females were egg positive. The distribution of positive individuals according to age group is shown in Supplementary Table 1.

### Accuracy analysis and POC-CCA test “trace” results

Evaluation of the POC-CCA test performance in relation to the RS revealed a sensitivity of 61.9%. In comparison with each of the parasitological tests, 16 KK slides and the HTX^®^ method, the sensitivity of the POC-CCA test decreased from 80.6% to 56.6%. Additional data regarding the accuracy analysis are described in Table 1.

**TABLE 1:** Comparison of the number of individuals detected with intestinal schistosomiasis by the reference standard, KK technique, and HTX in relation to positive individuals detected by the rapid urine test (POC-CCA).

POC-CCA	Reference Standard			KK 16S 3SA			HTX^®^		
	Positive	Negative	Total	Positive	Negative	Total	Positive	Negative	Total
**Positive**	39	40	79	25	54	79	30	46	79
**Negative**	24	114	138	6	132	138	23	115	138
**Total**	63	154	217	31	186	217	53	164	217
**Sensitivit**y	61.9% (95% *CI*: 49.5 - 72.9)			80.6% (95% *CI*: 63.7 - 90.8)			56.6% (95% *CI*: 43.3 - 69.0)		
**Specificity**	74.0% (95% *CI*: 66.6 - 80.3)			71.0% (95% *CI*: 64.1 - 77.0)			70.1% (95*% CI*: 62.7 - 76.6)		
**PPV**	49.4% (95% *CI*: 38.6 - 60.1)			31.6% (95% *CI*: 22.4 - 42.5)			38.0% (95% *CI*: 28.1 - 49.0)		
**NPV**	82.6% (95% *CI*: 75.4 - 88.0)			95.6% (95% *CI*: 90.8 - 98.0)			83.3% (95% *CI*: 76.2 - 88.6)		
**Kappa index**	0.33 (95% *CI*: 0.20 - 0.46)			0.31 (95% *CI*: 66.0 - 77.9)			0.22 (95% *CI*: 0.10 - 0.36)		
**Accuracy**	70.5% (95% *CI*: 64.1 - 76.2)			72.3% (95% *CI*: 0.20 - 0.42)			66.8% (95% *CI*: 60.3 - 72.7)		

**POC-CCA:** Point-of-care circulating cathodic antigen test; **PPV:** Positive predictive value; **NPV:** Negative predictive value; **RS:** composed of a total of 16 KK slides and 30 grams of fecal matter, examined by the HTX method; **16S 3SA:** Sixteen slides, twelve slides from the first sample, two slides from the second sample, and two slides from the third sample; **HTX:** Helmintex^®^ method.

**FIGURE 1:**
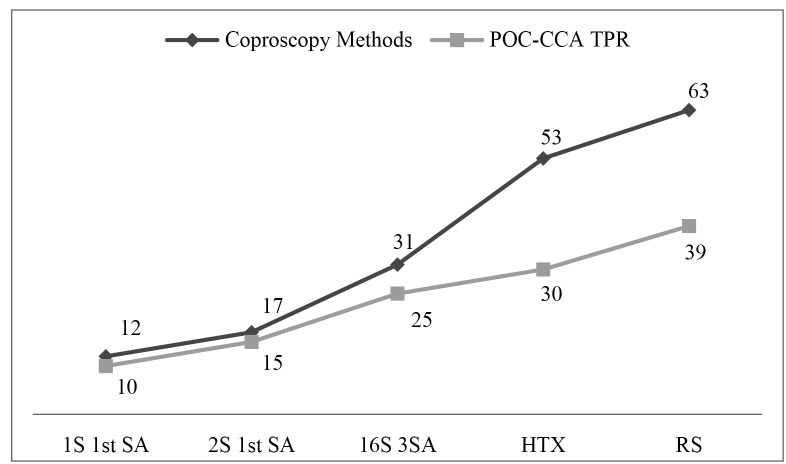
True positive results detected by the POC-CCA test confirmed by different coproscopic methods.**POC-CCA TPR:** Point of care circulating cathodic antigen true positive results; coproscopic methods: (**1S 1** ^st^ **SA:** One slide of the first sample; **2S 1** ^st^ **SA:** Two slides of the first sample; **16S 3SA:** Sixteen slides of three different samples, twelve slides of the first sample, two slides of the second sample and two slides of the third sample; **HTX** ^®^ **:** Helmintex^®^ method); **RS:** Reference standard

In the comparisons between the POC-CCA and RS test results, a total of 39 individuals were found to be egg positive. This number decreased to 25 and 30 egg positive individuals when the test was compared to each of the parasitological methods (e.g., the KK and HTX methods, respectively). More details are shown in Table 1, Table 2, and Figure 1.

**TABLE 2:** Number of individuals with intestinal schistosomiasis, as detected by one (1S 1st SA) or two (2S 1st SA) fecal thick smears and concordance and accuracy with the rapid urine test (POC-CCA).

POC-CCA	1S 1^st^ AS			2S 1^st^ AS		
	Positive	Negative	Total	Positive	Negative	Total
**Positive**	10	69	79	15	64	79
**Negative**	2	136	138	2	136	138
**Total**	12	205	217	17	200	217
**Sensitivity**	83.3% (95% *CI*: 55.2 - 95.3)			88.2% (95% *CI*: 65.6 - 96.7)		
**Specificity**	66.3% (95% *CI*: 59.6 - 72.4)			68.0% (95% *CI*: 61.2 - 74.1)		
**PPV**	12.6% (95% *CI*: 7.0 - 21.7)			19.0% (95% *CI*: 11.8 - 29.0)		
**NPV**	98.5% (95% *CI*: 94.9 - 99.6)			98.5% (95% *CI*: 94.9 - 99.6)		
**Kappa index**	0.13 (95% *CI*: 0.06 - 0.21)			0.21 (95% *CI*: 0.12 - 0.30)		
**Accuracy**	67.3% (95% *CI*: 60.8 - 73.1)			69.5% (95% *CI*: 63.2 - 75.3)		

**POC-CCA:** Point-of-care circulating cathodic antigen test; **1S 1** ^st^ **SA:** One slide of the first sample; **2S 1** ^st^ **SA:** Two slides of the first sample; **PPV:** Positive predictive value; **NPV:** Negative predictive value

Analysis of the POC-CCA test showed a total of 42 weak positive results out of 79 positive individuals detected, which were classified as “trace” results. Out of these 42 trace results, a total of 15 were classified as egg positive and 27 as egg negative when compared with the RS (Table 3).

**TABLE 3:** Positive and negative results determined by the reference standard in relation to the POC-CCA test results, considering trace results as negative.

POC-CCA	Reference standard		
	Positive	Negative	Total
**Positive**	23	14	37
**Negative**	40	140	180
**Total**	63	154	217

**POC-CCA:** Point-of-care circulating cathodic antigen test; Reference standard: composed of a total of 16 KK slides and 30 grams of fecal matter, examined by HTX.

### Parasitological and immunological results 30 days after treatment

Out of 63 positive individuals detected by the RS, only 53 continued in the evaluation 30 days after treatment. Out of the 53 participants, a total of 45 (81.1%) individuals had egg negative results, and 8 (18.9%) were still egg positive according to the RS. Using 16 slides, KK tests of different samples revealed no infected individuals. The HTX method detected a total of 8 (18.9%) egg-positive individuals after 30 days of treatment. A total of 5 (9.4%) egg-positive and 37 (69.8%) egg-negative individuals were detected when the POC-CCA test was used. Furthermore, 11 (20.7%) trace results were detected. Table 4 shows the relation between POC-CCA and the RS results after treatment.

**TABLE 4:** Concordance between POC-CCA results in relation to the reference standard, 16 slides from different samples and HTX results 30 days after treatment.

POC-CCA	RS			KK (16S 3SA)			HTX		
	Positive	Negative	Total	Positive	Negative	Total	Positive	Negative	Total
Positive	2	14 (11)*	16	0	16 (11)*	16	2	14 (11)*	16
**Negative**	6	31	37	0	37	37	6	31	37
**Total**	8	45	53	0	53	53	8	45	53

**RS:** Reference standard; **16S 3SA:** Sixteen slides, twelve slides from the first sample, two slides from the second sample and two slides from the third sample; **HTX:** Helmintex^®^ method. *Trace results classified as positive.

## DISCUSSION

Over the last 30 years, efforts made by the Brazilian Schistosomiasis Control Program have contributed to decreasing the positivity rate and individual parasite loads in endemic areas, consequently hampering *S. mansoni* egg detection by the KK method^4^ ^,^ ^9^. The use of two slides under these circumstances does not provide satisfactory diagnostic performance to proceed toward elimination of schistosomiasis as a public health problem^8^ ^,^ ^11^ ^-^ ^14^ ^,^ ^21^.

A study conducted by Lindholz *et al*. (2018)^17^ used three different diagnostic methods (the KK technique, HTX method, and POC-CCA test) and compared the tests performances among individuals with low parasite loads, where the HTX^®^ yielded more sensitive results than two KK slides.

Another Brazilian study used a RS combining 18 KK slides of three different stool samples, the saline gradient technique, and the HTX method in an endemic area in the northern part of Minas Gerais State^14^. The HTX method yielded better results than any combination of KK slides and much better results than the saline gradient technique.

Our study also demonstrated that the HTX method detected 22 positive results on more than 16 KK slides. On the other hand, the KK method confirmed a total of 9 positive individuals who had not been identified by the HTX method. Therefore, the combination of both methods was chosen as the RS because it maximizes the detection of egg positive samples.

Currently, the POC-CCA test demonstrated better results than two KK slides^20^. In a study conducted by Sousa *et al*. (2019)^21^ in a low prevalence area with low individual parasite loads, it was noticed that the POC-CCA test had similar rates of detection of infected individuals when compared with 9 slides from a single stool sample or 6 slides from 3 different samples. However, the increase in sensitivity had shown an improvement of performance of the POC-CCA test with an increase in the amount of feces analyzed. Thus, it was concluded that it was necessary to improve the RS to better evaluate the CCA test.

The present study indicated an increase in the sensitivity of the POC-CCA test when the coproscopic techniques were combined. Therefore, it is notable that a total of 39 true positives were detected by the POC-CCA test when compared with our RS. This value yielded a better detection rate than 16 KK slides from three different stool samples, which identified 31 positives (Figure 1). This is a higher rate than that reported by Sousa *et al*. (2019)^21^.

Studies carried out in moderate and high prevalence areas showed that the POC-CCA test is more sensitive than the KK method and can be used for screening and geographical mapping of *S*. *mansoni* infections. A sensitivity rate of up to 99.5% was reached when a latent class analysis model was used^25^. However, the sensitivity of the POC-CCA test was compromised when it was applied in a low prevalence area with low parasite load. Oliveira *et al.* (2018)^14^ and Lindholz *et al.* (2018)^17^ described a decreased sensitivity of the POC-CCA test when the parasite load was below 100 EPG. 

Our study showed that the highest concordance was reached when compared with our established RS. In this context, if only the immunochromatographic test would be used for the diagnosis of an active *S. mansoni* infection, 40 positive individuals out of a total of 79 would show false positive results in comparison to the RS, and 24 egg positive individuals would not be detected by POC-CCA. This means that approximately 50% of them would be treated unnecessarily. In contrast, by using only two KK slides and comparing them to the RS, a total of 46 positive results would be missed.

A total of 16 (25.4%) egg-positive individuals were not detected when the “trace” result was classified as a negative result. This result revealed that the POC-CCA test detects more infected individuals when “trace” results are classified as positive results, as also described by Prada *et al*. 2018^26^. In contrast, when “trace” results were considered negative, the POC-CCA test showed better performance in the detection of true negative results. At this point, the interpretation of “trace” results may be ambiguous, as described by Clements *et al*. (2017)^27^.

In our study, the area of prevalence may reach a different classification, depending on the applied diagnostic method, and would result in a completely different recommended strategy for the treatment of the population^9^. Our data reinforce the need to associate different diagnostic tools to improve the detection of *S. mansoni* in individuals with low parasitic load, as recommended by Bezerra *et al*. (2018)^28^.

Thirty days after treatment, all the KK results were negative, and only HTX indicated egg positivity. The scenarios observed in this study and other publications evaluating the presence of *Schistosoma* infection after treatment demonstrated that infections with low parasite loads were more frequently detected when a larger amount of feces was analyzed^15^ ^,^ ^29^ ^,^ ^14^ ^,^ ^17^.

The performance of the POC-CCA test 30 days after treatment shows poor detection of positive individuals. Out of 14 false positive results, 11 were “trace” results and classified as positive. The high frequency of false positive results could be explained by a small number of surviving worm couples, which stopped releasing eggs because of the damaging effects of praziquantel or by surviving juvenile forms of the parasite that are not susceptible to praziquantel treatment. However, more studies need to be conducted to elucidate the mechanism underlying this observation. In relation to the loss of 6 (75.0%) egg-positive individuals, as detected by RS after treatment, would be the possibility of prolonged release of eggs, even after killing adult worms, which would result in a decrease in circulating antigens and in human blood within 2 to 3 weeks after treatment^19^. However, the present study did not seek to elucidate this mechanism.

## CONCLUSION

In summary, our data indicated that the POC-CCA test has potential as an auxiliary tool for the diagnosis of *S. mansoni* infections, revealing better results than 16 KK slides from three different stool samples. However, the immunochromatographic test was found not to be a specific and sufficiently sensitive tool to verify the cure rate after praziquantel treatment. This is in discordance with the findings of Prada *et al*. (2018)^27^, which suggested the CCA test as a better predictor of prevalence after treatment. In relation to the performance of the KK technique, this study revealed that this method lacks accuracy due to the decrease in the individual parasite load in a previously treated population. In contrast, the HTX method revealed solid results as a potential alternative and additional tool for the evaluation of cure after treatment of *S. mansoni* infections.

## References

[1] World Health Organization (WHO) (2013). Schistosomiasis: progress report 2001-2011 and strategic plan
2012-2020.

[2] World Health Organization (WHO) (2016). Schistosomiasis and soil-transmitted helminths: number of people treated
in 2015.

[3] World Health Organization (WHO) (2019). Schistosomiasis: Key facts.

[4] Noya O, Katz N, Pointier JP, Theron A, Noya BA (2015). Schistosomiasis in America. PLoS Negl Trop Dis.

[5] Katz N (2018). Inquérito nacional de prevalência da esquistossomose mansoni e
geo-helmintoses (2010-2015).

[6] World Health Organization (WHO) (2012). Sixty-fifth world health assembly.

[7] Brasil (2017). Programa de Vigilância e Controle da Esquistossomose.

[8] De Vlas SJ, Gryseels B (1992). Underestimation of Schistosoma mansoni
prevalences. Parasitol Today.

[9] Ministério da Saúde (MS). Secretaria de Vigilância em Saúde (2014). Vigilância da Esquistossomose mansoni: diretrizes técnicas.

[10] Katz N, Chaves A, Pellegrino J (1972). A simple device for quantitative stool thick-smear technique in
Schistosomiasis mansoni. Rev Inst Med Trop Sao Paulo.

[11] Gryseels B, De Vlas SJ (1996). Worm burdens in Schistosome infections. Parasitol Today.

[12] Enk M.J, Lima AC, Massara CL, Coelho PM, Schall VT (2008). A combined strategy to improve the control of Schistosoma mansoni
in areas of low prevalence in Brazil. Am J Trop Med Hyg.

[13] Sousa SRM, Carvalho AQ, Cardoso JFN, Coelho PMZ, Geiger SM, Enk MJ (2017). Schistosomiasis in the Amazon region: is the current diagnostic
strategy still appropriate?. Rev Soc Bras Med Trop.

[14] Oliveira WJ, Magalhães FC, Elias MAS, Castro VN, Favero V, Lindholz CG (2018). Evaluation of diagnostic methods for the detection of intestinal
schistosomiasis in endemic area with low parasite loads: Saline gradient,
Helmintex, Kato-Katz and rapid urine test. PLoS Negl Trop Dis.

[15] Teixeira CF, Neuhauss E, Ben R, Romanzini J, Graeff-Teixeira C (2007). Detection of Schistosoma mansoni Eggs in Feces through their
Interaction with Paramagnetic Beads in a Magnetic Field. PLoS Negl Trop Dis.

[16] Favero V, Candido RRF, De Marco Verissimo C, Jones MK, St Pierre TG, Lindholz CG (2017). Optimization of the Helmintex meth­od for schistosomiasis
diagnosis. Exp Parasitol.

[17] Lindholz CG, Favero V, Verissimo CM, Candido RRF, de Souza RP, dos Santos RR (2018). Study of diagnostic accuracy of Helmin­tex, Kato-Katz, and
POC-CCA methods for diagnosing intestinal schistosomiasis in Candeal, a low
intensity transmission area in northeastern Brazil. PLoS Negl Trop Dis.

[18] Silva-Moraes S, Shollenberger LM, Siqueira LMV, Castro-Borges W, Harn DA, Grenfell RFQ, Rabello ALT, Coelho PMZ (2019). Diagnosis of Schistosoma mansoni infections: what are the choices
in Brazilian low-endemic areas?. Mem Inst Oswaldo Cruz.

[19] Rapid medical Diagnostics (2015). For qualitative detection of: Bilharzia (Schistosomiasis).

[20] Lamberton PHL, Kabatereine NB, Oguttu DW, Fenwick A, Webster JB (2014). Sensitivity and specificity of multiple Kato-Katz thick smears
and a Circulating Cathodic Antigen test for Schistosoma mansoni diagnosis
pre- and post-repeated-praziquantel treatment. PLoS Negl Trop Dis.

[21] Sousa SRM, Dias IHL, Fonseca ALS, Contente BR, Nogueira JFC, Oliveira TNC (2019). Concordance of the point-of-care circulating cathodic antigen
test for the diagnosis of intestinal schistosomiasis in a low endemicity
area. Infect Dis Poverty.

[22] Colley DG, Binder S, Campbell C, King CH, Tchuem Tchuenté LA, N'Goran EK (2013). A Five-Country Evaluation of a Point-of-Care Circulating Cathodic
Antigen Urine Assay for the Prevalence of Schistosoma
mansoni. Am J Trop Med Hyg.

[23] Instituto Brasileiro de Geografia e Estatística (IBGE) (2017). Turiaçú, Maranhão. Censo Demográfico 2017.

[24] Landis JR, Koch GG (1977). The Measurement of Observer Agreement for
Categorical. Biometrics.

[25] Fuss A, Mazigo HD, Tappe D, Kasang C, Mueller A (2018). Comparison of sensitivity and specificity of three diagnostic
tests to detect Schistosoma mansoni infections in school children in Mwanza
region, Tanzania. PLoS Negl Trop Dis.

[26] Prada JM, Touloupou P, Adriko M, Tukahebwa EM, Lamberton PHL, Hollingsworth TD (2018). Understanding the relationship between egg- and antigen-based
diagnostics of Schistosoma mansoni infection pre- and post-treatment in
Uganda. Parasite & Vectors.

[27] Clements MN, Donnelly CA, Fenwick A, Kabatereine NB, Knowles SCL, Meite A (2017). Interpreting ambiguous `trace' results in Schistosoma mansoni CCA
Tests: Estimating sensitivity and specificity of ambiguous results with no
gold standard. PLoS Negl Trop Dis.

[28] Bezerra FSM, Leal JKF, Sousa MS, Pinheiro MCC, Júnior ANR, Silva-Moraes V, Katz N (2018). Evaluating a point-of-care circulating cathodic antigen test
(POC-CCA) to detect Schistosoma mansoni infections in a low endemic area in
north-eastern Brazil. Acta Trop.

[29] Caldeira K, Teixeira CF, Silveira MB, Fries LCC, Romanzini J, Bittencourt HR (2012). Comparison of the Kato-Katz and Helmintex methods for the
diagnosis of schistosomiasis in a low-intensity transmission focus in
Bandeirantes, Paraná, southern Brazil. Mem Inst Oswaldo Cruz.

