# Supplementary figures and images for: The use of the circulating cathodic antigen (CCA) urine cassette assay for the diagnosis and assessment of cure of Schistosoma mansoni infections in an endemic area of the Amazon region
Source: Rev Soc Bras Med Trop. 2020 Sep 25;53:e20190562. doi: 10.1590/0037-8682-0562-2019 (PMC7523523; doi:10.1590/0037-8682-0562-2019)

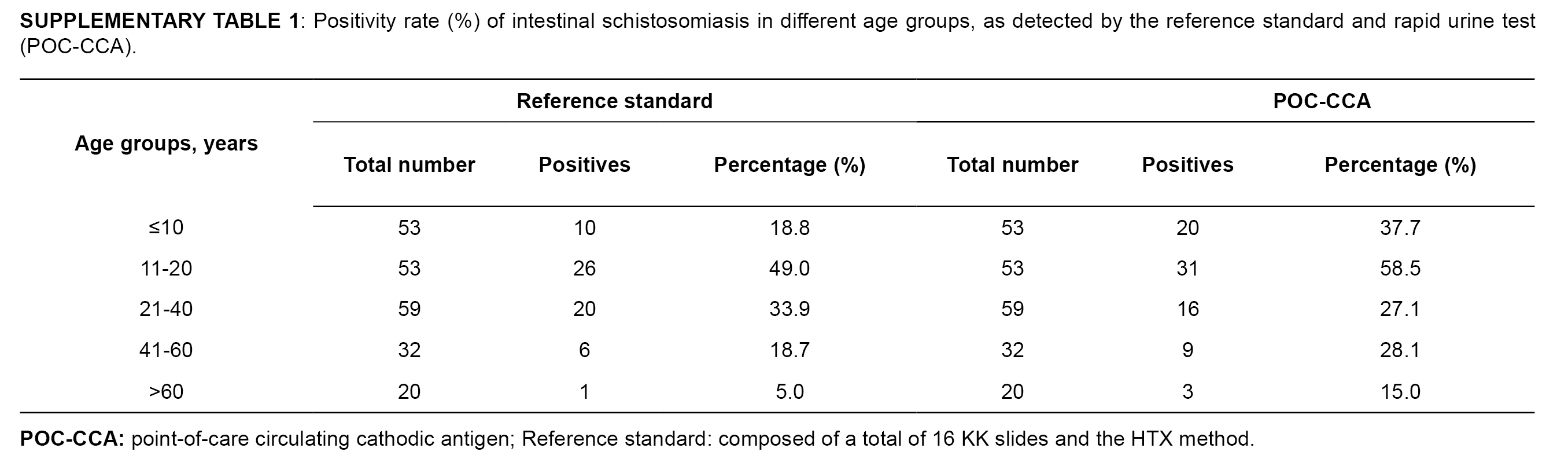

Supplement: Supplementary file 1 [file 1678-9849-rsbmt-53-e20190562-suppl1.jpg]
